# Tracheal Actinomycosis Presenting as a Pseudotumor: A Case Report

**DOI:** 10.7759/cureus.113442

**Published:** 2026-07-27

**Authors:** Marcela Velásquez-Salazar, Carlos Darío Aguilar Díaz

**Affiliations:** 1 Surgery, University of Caldas, Manizales, COL; 2 Pulmonology, University of Caldas, Manizales, COL

**Keywords:** actinomycosis, airway obstruction, pseudotumor, tracheal actinomycosis, tracheal pseudotumor

## Abstract

Actinomycosis is a rare infection that can easily be confused with a neoplasm when it forms mass-like lesions. We report the case of a 21-year-old male patient who presented with dyspnea, cough, and stridor. Imaging studies revealed a tracheal mass obstructing the airway. Bronchoscopy confirmed the presence of the mass, and tissue samples were obtained for pathological examination. Histopathological examination revealed actinomycosis. Following treatment with ampicillin/sulbactam, surgical resection, and tracheostomy, the patient made a satisfactory recovery. This case highlights the importance of considering actinomycosis in the differential diagnosis of unexplained airway masses that mimic neoplasms.

## Introduction

Actinomycosis is a rare infectious disease caused by *Actinomyces *spp., which are Gram-positive anaerobic filamentous bacilli [1,2]. The diagnosis of this condition is challenging because it requires a high index of suspicion and invasive procedures to obtain adequate samples for identification of the organism, either through prolonged anaerobic cultures or histopathological findings characterized by aggregates of mycelial fragments with the characteristic appearance of sulfur granules that stain positive with periodic acid-Schiff (PAS) [2,3]. The most common anatomical sites of infection include cervicofacial, pelvic, and thoracic actinomycosis. Other unusual manifestations have been reported involving the genital tract, skin, bloodstream, and upper respiratory tract [3,4]. Tracheal lesions caused by actinomycosis are very rare [3,4]. Furthermore, several case reports have described actinomycosis as a potential mimic of neoplastic processes [5,6]. The purpose of this study is to describe a case of tracheal actinomycosis.

## Case presentation

**Figure 1:**
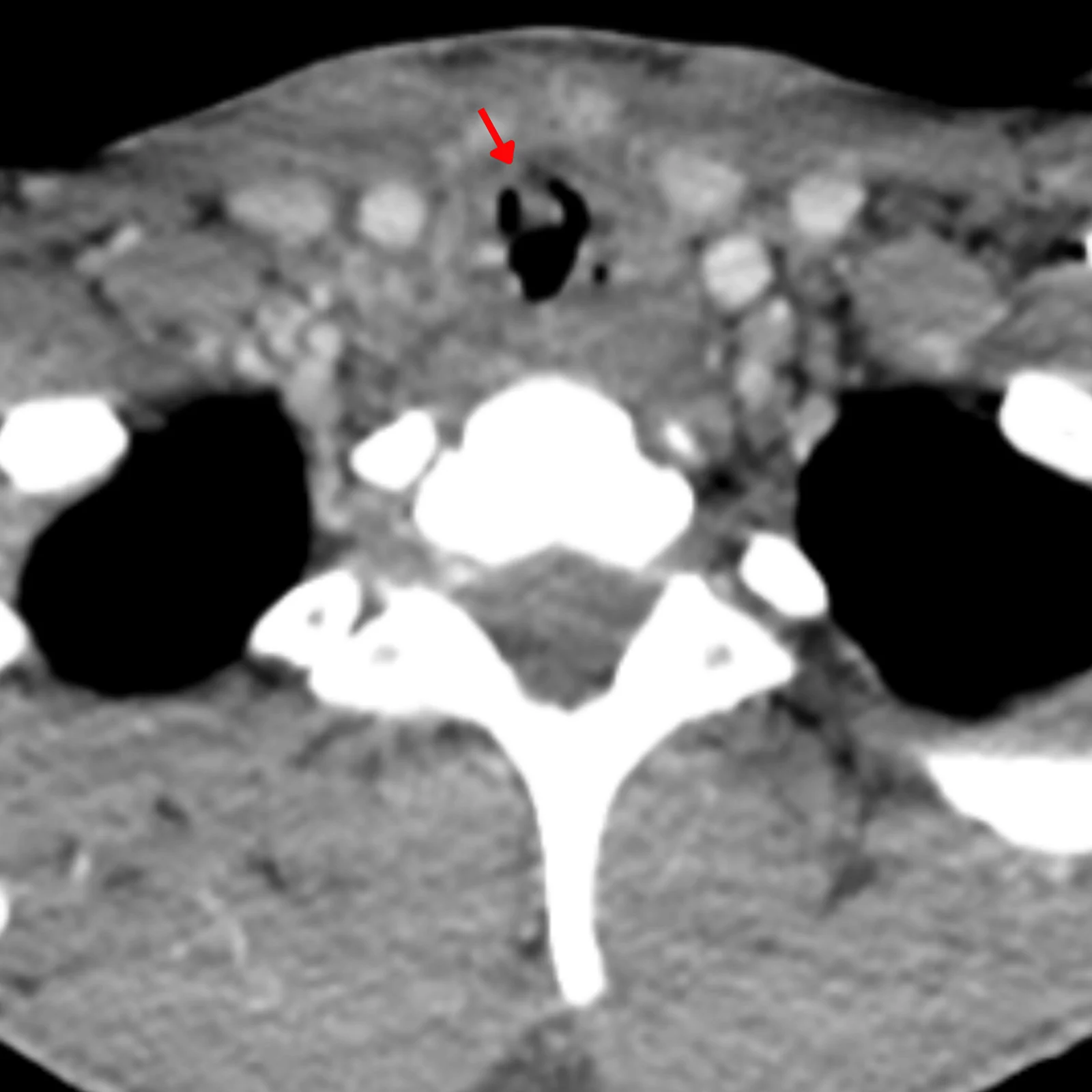
Contrast-enhanced CT scan of the neck showing a stenotic lesion located superior to the cricoid cartilage (red arrow)

In January 2026, a 21-year-old Colombian man with a history of type 1 diabetes mellitus presented with a three-day history of dyspnea, dry cough, and audible stridor. On admission to a clinic in Manizales, Colombia, he was tachycardic, normotensive, and tachypneic, with normal oxygen saturation. Physical examination revealed signs of respiratory distress, including supraclavicular retractions. No cervical masses or lymphadenopathy were identified, and pulmonary auscultation demonstrated preserved vesicular breath sounds bilaterally. Initial laboratory testing revealed leukocytosis (15.000 cells/uL) and neutrophilia (13.000 cells/uL). Blood glucose levels were unmeasurably elevated (628 mg/dL), arterial blood gas analysis demonstrated high anion gap metabolic acidosis, and electrolyte levels were within normal limits. Diabetic ketoacidosis was diagnosed, prompting admission to the intensive care unit, and the patient was admitted to the intensive care unit for management of both diabetic ketoacidosis and upper airway obstruction. A contrast-enhanced computed tomography scan of the neck demonstrated a stenotic lesion located superior to the cricoid cartilage (Figure 1).

**Figure 2:**
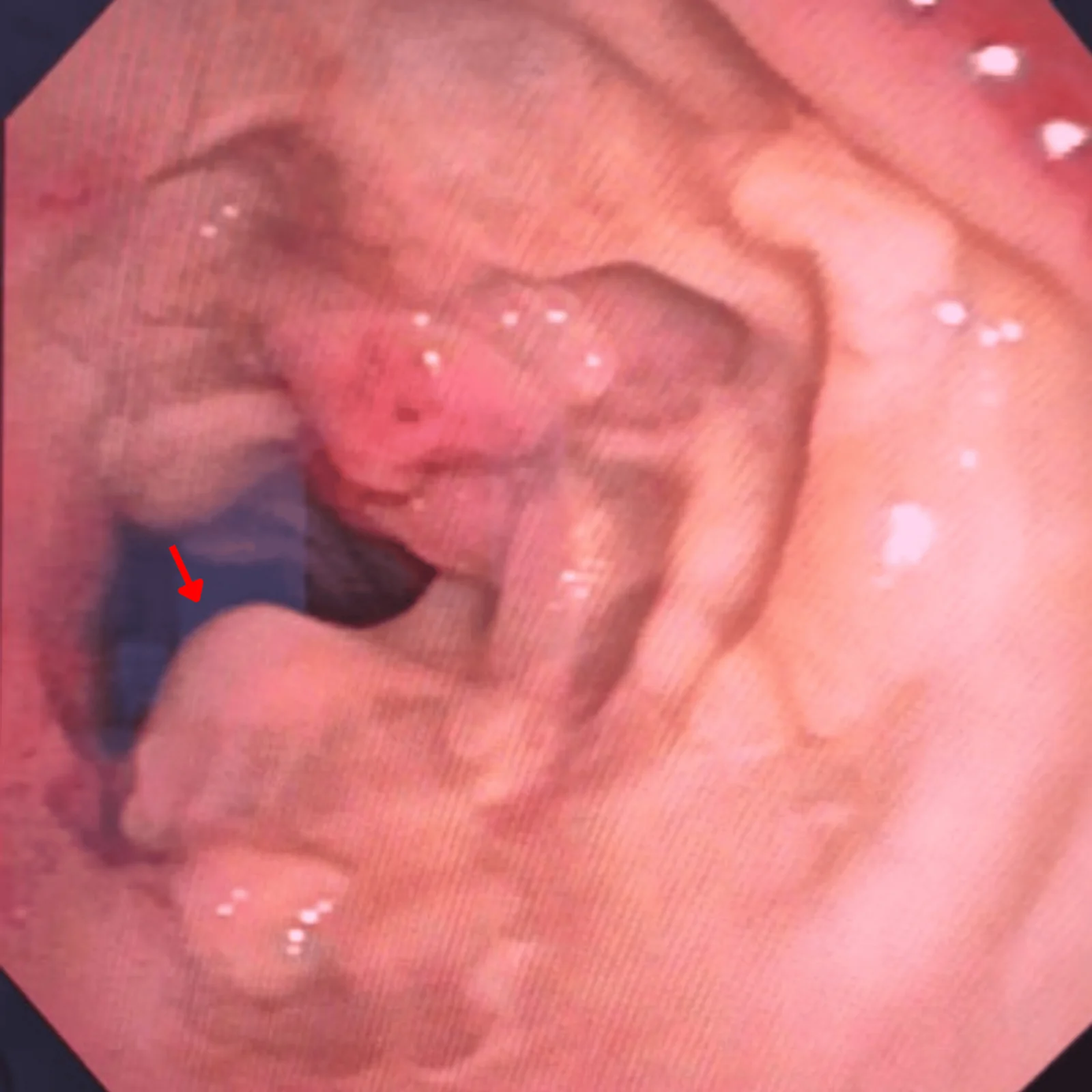
Flexible bronchoscopy demonstrating a smooth, pink tracheal lesion causing partial airway obstruction (red arrow)

**Figure 3:**
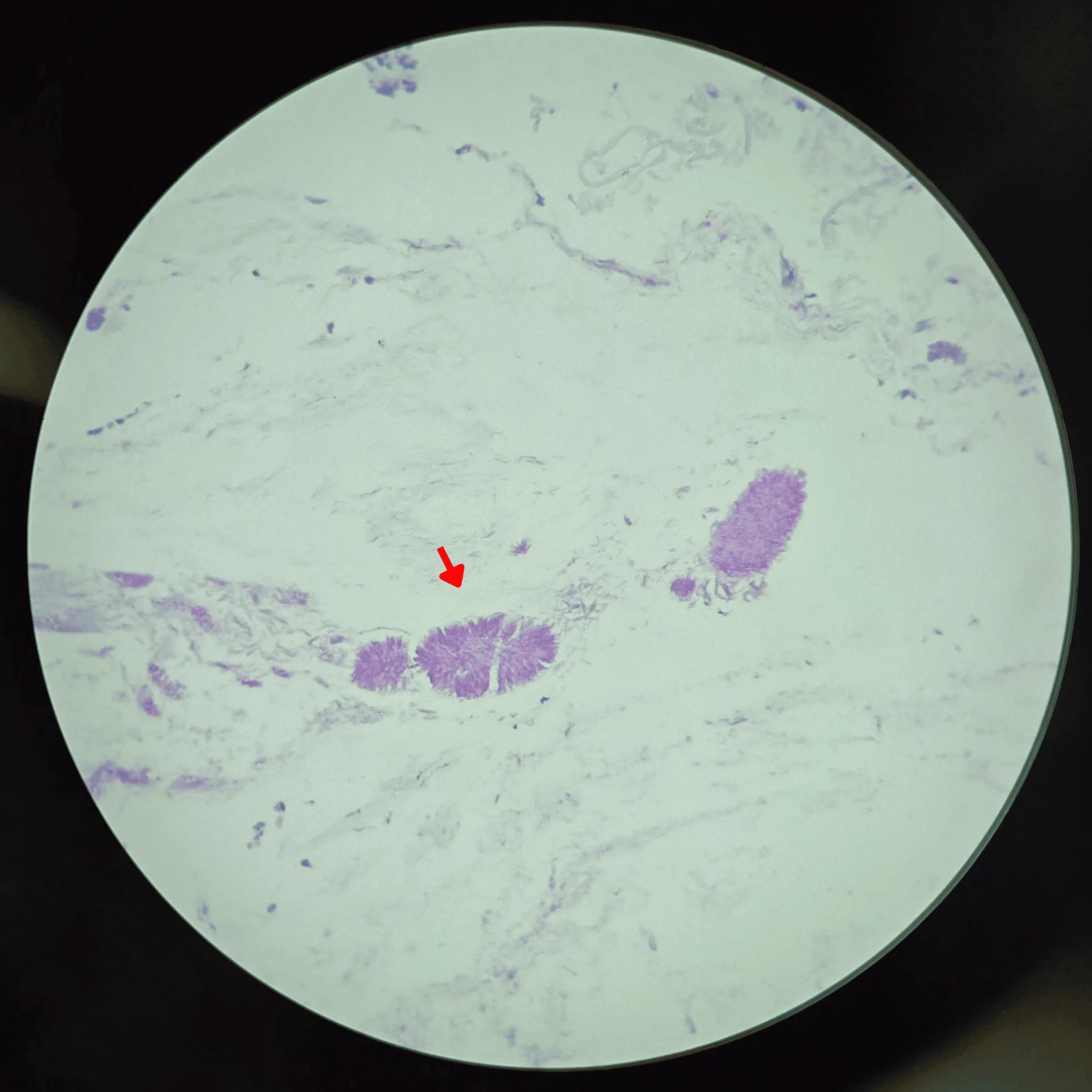
Histopathological specimen demonstrating aggregates of filamentous organisms consistent with actinomycosis (red arrow)

Pulmonology consultation was obtained two days later, and flexible bronchoscopy was performed (Figure 2), which revealed a smooth, pink tracheal mass that was firm, causing approximately 25% luminal obstruction. Left vocal cord paralysis and a healing ulcerative lesion were also observed. Based on these findings, treatment with ampicillin/sulbactam was initiated. Biopsy specimens were obtained and demonstrated definitive histopathological evidence of actinomycosis (Figure 3).

Subsequently, the patient was referred to thoracic surgery, where surgical resection of the lesion and tracheostomy were performed. Histopathological examination of the surgical specimen no longer demonstrated evidence of actinomycosis, which demonstrates successful antibiotic treatment.

## Discussion

Actinomycosis is caused by a microorganism generally considered part of the normal human microbiota. Consequently, disease is uncommon in immunocompetent individuals. It is more frequently associated with conditions that compromise host immunity, including chronic bronchitis, emphysema, cervicofacial trauma, periodontal procedures, and diabetes mellitus [2], as observed in the present case. Tracheal actinomycosis is an exceptionally uncommon manifestation, and only limited literature is available regarding this localization [1,3]. Reported cases are frequently misdiagnosed as abscesses, tuberculosis, or malignant neoplasms because of overlapping clinical features such as dyspnea, cough, and hemoptysis. In addition, the mass had a similar macroscopic appearance during endoscopic evaluation, as demonstrated in our patient [3-7]. Furthermore, in this case, the patient presents with left vocal cord paralysis; this is explained by the compression of the recurrent laryngeal nerve by the mass [3-7]. In addition to tracheal pseudotumors, pseudotumoral lesions caused by actinomycosis have been described in other anatomical locations, including the gastrointestinal tract, particularly the colon, and the pelvis, especially among women using intrauterine devices. Furthermore, one case report described the simultaneous occurrence of pulmonary actinomycosis and squamous cell carcinoma in the same patient [8,9].

## Conclusions

Actinomycosis can present with clinical and radiological features that closely mimic neoplastic lesions in various organs. This case highlights a tracheal pseudotumor diagnosed by histopathological examination of a bronchoscopic biopsy specimen. Clinicians should therefore consider actinomycosis in the differential diagnosis of tracheal or endobronchial masses of uncertain origin.
